# Comparability of Mixed IC_50_ Data – A Statistical Analysis

**DOI:** 10.1371/journal.pone.0061007

**Published:** 2013-04-16

**Authors:** Tuomo Kalliokoski, Christian Kramer, Anna Vulpetti, Peter Gedeck

**Affiliations:** Global Discovery Chemistry, Novartis Institutes for Biomedical Research, Basel, Switzerland; University of Bologna & Italian Institute of Technology, Italy

## Abstract

The biochemical half maximal inhibitory concentration (IC_50_) is the most commonly used metric for on-target activity in lead optimization. It is used to guide lead optimization, build large-scale chemogenomics analysis, off-target activity and toxicity models based on public data. However, the use of public biochemical IC_50_ data is problematic, because they are assay specific and comparable only under certain conditions. For large scale analysis it is not feasible to check each data entry manually and it is very tempting to mix all available IC_50_ values from public database even if assay information is not reported. As previously reported for K_i_ database analysis, we first analyzed the types of errors, the redundancy and the variability that can be found in ChEMBL IC_50_ database. For assessing the variability of IC_50_ data independently measured in two different labs at least ten IC_50_ data for identical protein-ligand systems against the same target were searched in ChEMBL. As a not sufficient number of cases of this type are available, the variability of IC_50_ data was assessed by comparing all pairs of independent IC_50_ measurements on identical protein-ligand systems. The standard deviation of IC_50_ data is only 25% larger than the standard deviation of K_i_ data, suggesting that mixing IC_50_ data from different assays, even not knowing assay conditions details, only adds a moderate amount of noise to the overall data. The standard deviation of public ChEMBL IC_50_ data, as expected, resulted greater than the standard deviation of in-house intra-laboratory/inter-day IC_50_ data. Augmenting mixed public IC_50_ data by public K_i_ data does not deteriorate the quality of the mixed IC_50_ data, if the K_i_ is corrected by an offset. For a broad dataset such as ChEMBL database a K_i_- IC_50_ conversion factor of 2 was found to be the most reasonable.

## Introduction

Public collections of IC_50_ data (the half maximal inhibitory concentrations of ligands on their protein targets) represent a wealth of knowledge on bioactivity with growing importance. One of the major databases of public bioactivities for small molecules is ChEMBL, [1] which currently contains roughly three times more IC_50_ values than K_i_ values. It has been shown that the gap between the number of IC_50_ and K_i_ values is still increasing. [2] Proper usage of IC_50_ data facilitates the development of useful methods for drug discovery. Examples of such applications are the global mapping of pharmacological space by Paolini and co-workers, [3] the Similarity Ensemble Approach (SEA), [4] the Bayesian models for adverse drug reactions by Bender and coworkers, [5] the models used for polypharmacological optimization by Hopkins et al., [6] and the kinome-wide activity modeling studies by Schuerer and Muskal. [7] These methods can be used to predict off-target effects based on heterogeneous public activity data and chemical similarity analysis. Usually, public off-target toxicity models like human Ether-à-go-go-Related Gene (hERG) [8] and cytochrome P450 (CYP) models [9], [10] are based and validated on mixed public IC_50_ data, since there is not enough public data available that originates from one single assay.

In contrast to K_i_ values, IC_50_ data is assay specific. For the simplest typical case of competitive monosubstrate enzyme inhibition, K_i_ can be calculated from the IC_50_ according to the Cheng-Prusoff equation:
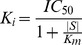
where |S| is the substrate concentration and K_m_ is the Michaelis-Menten constant of the substrate. [11] Under the same assay conditions the measured IC_50_ of same inhibitor or two different inhibitors (1 and 2 below) with the same mechanism of action can be compared as



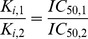



The problem is that assay details are not reported in public bioactivity databases. Recently, Zdrazil et al. analyzed human P-glycoprotein bioassay data from the ChEMBL and TP-search databases. [12] They explore the ability of these data, determined in different assays, to be combined with each other. Their study indicates that for inhibitors of human P-glycoprotein this is possible under certain conditions: i.e., data coming from the same type of assay, same cell lines, and also same fluorescent or radiolabeled substrates with overlapping binding sites. However they point out that it is currently not possible to extract such data in automated fashion from the current public databases. Effort in annotating assay details would increase the capabilities of safe data integration thus increasing the usefulness of those huge data repositories freely available.

In this manuscript we report an estimate of the error introduced by mixing public IC_50_ data from different labs and how this can affect the capability of drawing scientifically sound conclusions from such data. By using the same statistical technique that we have previously introduced to determine the experimental uncertainty of heterogeneous public K_i_ data [13] we analyze the variability of all pairs of biochemical IC_50_ measurements on the same protein-ligand system independently of assay details.

In the following, we first describe our attempts in extracting a set of at least ten IC_50_ values from ChEMBL that have independently been measured in two comparable assays. Since all sets of identified measurements turn out to be not independent or otherwise faulty, we analyze the standard deviation of all truly independent pairs of IC_50_ values available from ChEMBL. Dubious entries and filters used to spot and remove faulty entries are described in detail. For the remaining pairs of measurements, the original publications of protein-ligand systems showing various ranges of IC_50_ differences were inspected in order to gain an impression of which activity differences are due to database errors and which activity differences are due to the variations in assay conditions. We then fitted a Gaussian distribution to the distribution of IC_50_ differences to estimate the standard deviation of valid pairs of independent IC_50_ measurements. By comparing the IC_50_ standard deviation to the equivalent K_i_ standard deviation, we can estimate the variability of heterogeneous IC_50_ data. The average difference between K_i_ and IC_50_ values and their correlation are assessed. Moreover the effect of mixing K_i_ and IC_50_ values in order to enlarge the data size was evaluated. Lastly, we analyze whether the variability of IC_50_ values depends on simple ligand properties such as molecular weight (MW) and the calculated octanol –water partition coefficient (logP).

## Materials and Methods

### Dataset Preparation

All measurements were extracted for the ChEMBL database version 14. It is the currently largest public database with bioactivities extracted from the literature. BindingDB [14] is similar in size, but has a significant overlap with ChEMBL with most of the values being copied from ChEMBL.

The raw data was filtered in order to remove erroneous entries as described earlier. [13] Generally, all analyses presented here are based on multiple affinity measurements of the same protein-ligand system. The filtering steps were the following:

1. Remove all data from reviews, since this is not original data.
2. Remove all unclear measurements (i.e. Unit not M, mM, µM, nM, pM, fM; qualified values (“<” or “>”); extremely high (pActivity >15) or extremely low (pActivity <2) values).
3. Remove younger entry for exactly the same value reported twice (younger paper cites older paper).
4. Remove younger entry for very close values reported twice (difference in pActivity <0.02: younger paper cites older paper and rounds).
5. Remove both entries if their difference is exactly 3, 6, or 9. These are citations with unit-conversion errors.
6. Remove entries for which the authors could not be extracted from PubMed.
7. Only keep pairs where the name overlap of the authors is zero to make sure that measurements are from different laboratories.

After each step, protein-ligand systems that had only one measurement entry left (singletons) were removed. All affinity were converted to their negative logarithm pActivity (e.g. pIC_50_ or pK_i_) with M^−1^ as base unit (e.g. 1 µM is converted to 6 [log Activity units]).

In ChEMBL a confidence score is available for each bioactivity entry. According to the ChEMBL homepage, a confidence score of nine is the highest, a confidence score of four or more indicates a biochemical measurement and a confidence score below four indicates a cellular measurement. For the IC_50_ analysis, two sets of data were generated: Set1 contains all data with a confidence score of four and more, Set2 contains data with the highest confidence score nine only. Since it turned out that there is no difference in variability between Set1 and Set2, here we only report results for Set1.

From the initially available 616.555 IC_50_ values with confidence score greater or equal to four 10.895 IC_50_ values for 3.480 Protein/Ligand systems remained, yielding 20.356 pairs of independent measurements. Overall, the number of both protein/ligand systems and individual IC_50_ data points available for comparisons has been reduced by 94% and 93%. The filtering statistics is shown in Table 1.

**Table 1:** Filtering statistics for extracting independent pairs of IC_50_ measurements on identical systems.

Filter	# protein/ligand systems remaining	# IC_50_ data points remaining
Systems with multiple measurements only	54.505	137.043
Remove multiple values from identical publications	18.804	85.705
Remove exact duplicate values	8.387	33.187
Remove pairs with unit errors	8.141	22.770
Remove duplicates with rounding errors	7.263	19.487
Remove unrealistic values	7.228	19.383
Remove pairs with overlapping authors	3.480	10.895

### Metrics for Evaluating the Distribution of Errors

We analyze the distribution of the differences between two affinity measurements on the same protein-ligand system using the Standard Deviation (σ), the Mean Unsigned (Absolute) Error (MUE), the Median Unsigned Error (M_ed_UE) the squared Pearson’s correlation coefficient (R^2^_pearson_ = R^2^). They are defined as
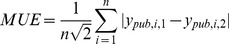


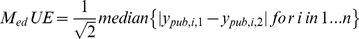


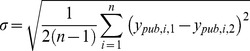


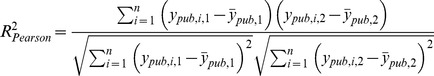


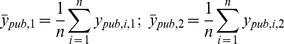
with n being the number of pairs of measurements considered, y_pub,i,1_ and y_pub,i,2_ being the two published values of pair i and _pub_ is the average of all measured values. If more than two measurements are available for a given protein-ligand system, all possible pairs are generated. The order of y_pub,i,1_ and y_pub,i,2_ has to be scrambled in order to not bias the calculation of *R^2^_Pearson_* and σ. As we have shown earlier, [13] MUE, M_ed_UE and σ calculated from pairs of measurements are overestimated by a factor of √2. Therefore MUE, M_ed_UE and σ calculated from pairs of measurements were divided by √2.

Raw data was extracted from ChEMBL14 using MySQL statements. Filtering and pairing of measurements were done using Python 2.7. The statistical analysis was carried out using R version 2.15.1. [15] All R-, Python- and MySQL-scripts used including detailed instructions on how to repeat the work can be found in the Archive S1.

## Results

In order to assess the comparability of IC_50_ values, we first extracted all series of compounds that have been measured against the same protein target in two independent assays from whole ChEMBL. There were twelve series of ten or more compounds whose activity on the same target has been measured in different assays. An overview of the different series is given in Supporting Information (Table S1, Text S1–S2 and Figures S1–S2). However, eleven out of twelve series had overlapping authors and the single independently measured series was incorrectly annotated into the database.

Since it is not possible to find independently measured sets of at least ten IC_50_ values for the same target, the IC_50_ variability was determined differently. In the following, we analyze the IC_50_ data using an approach that we have previously introduced for analyzing the reproducibility of heterogeneous K_i_ data. All pairs of identical protein-ligand systems with independently measured IC_50_ values were extracted from ChEMBL and the variability of the differences between the pairs of measurements was calculated.

**Figure 1:**
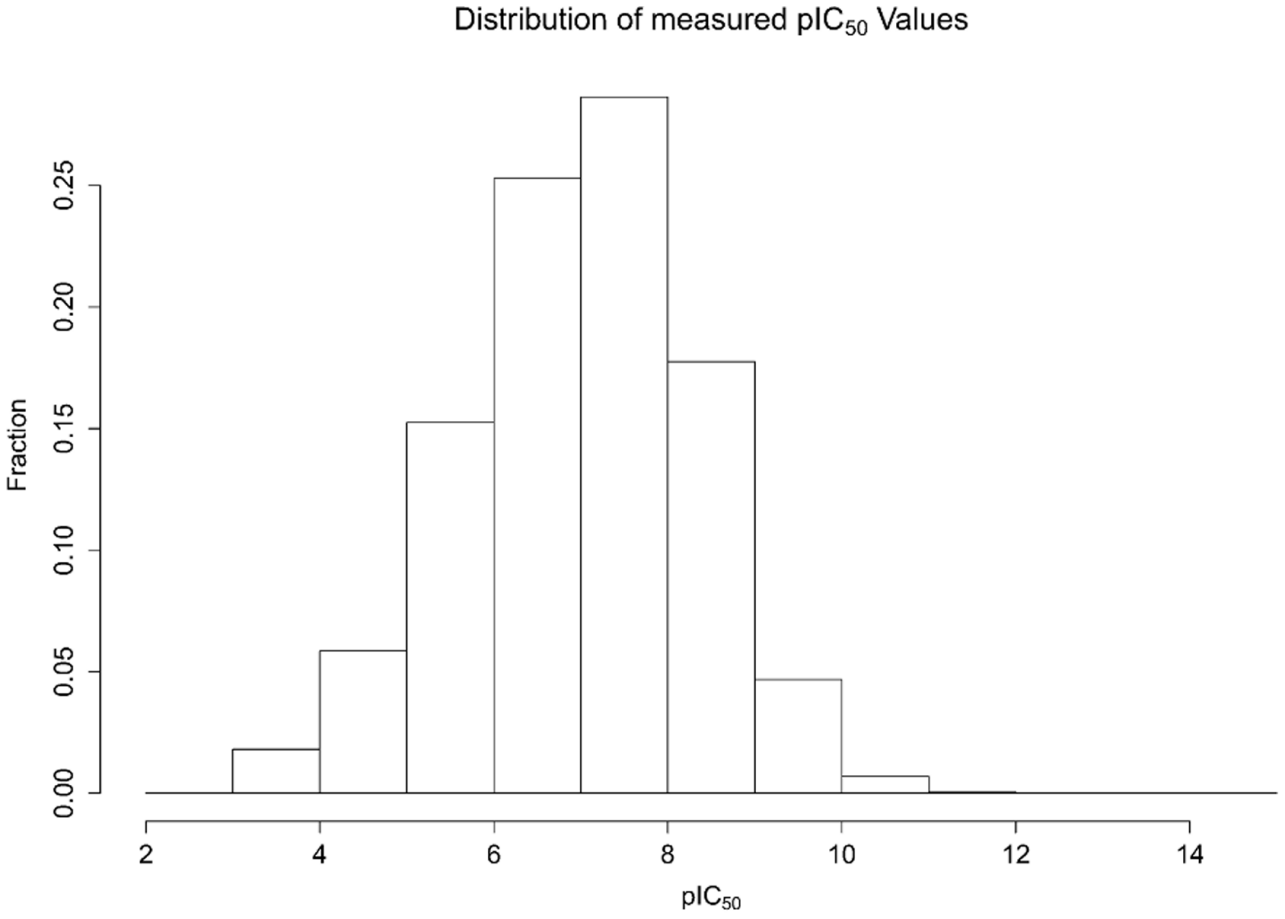
Distribution of the 9.465 pIC_50_ values for protein-ligand systems with independent multiple measurements.

The distribution of pIC_50_ values is shown in Figure 1. The distribution of measured values is slightly skewed to the left with a maximum of roughly 30% of all pIC_50_ values reported between 7.0 and 8.0.

**Figure 2:**
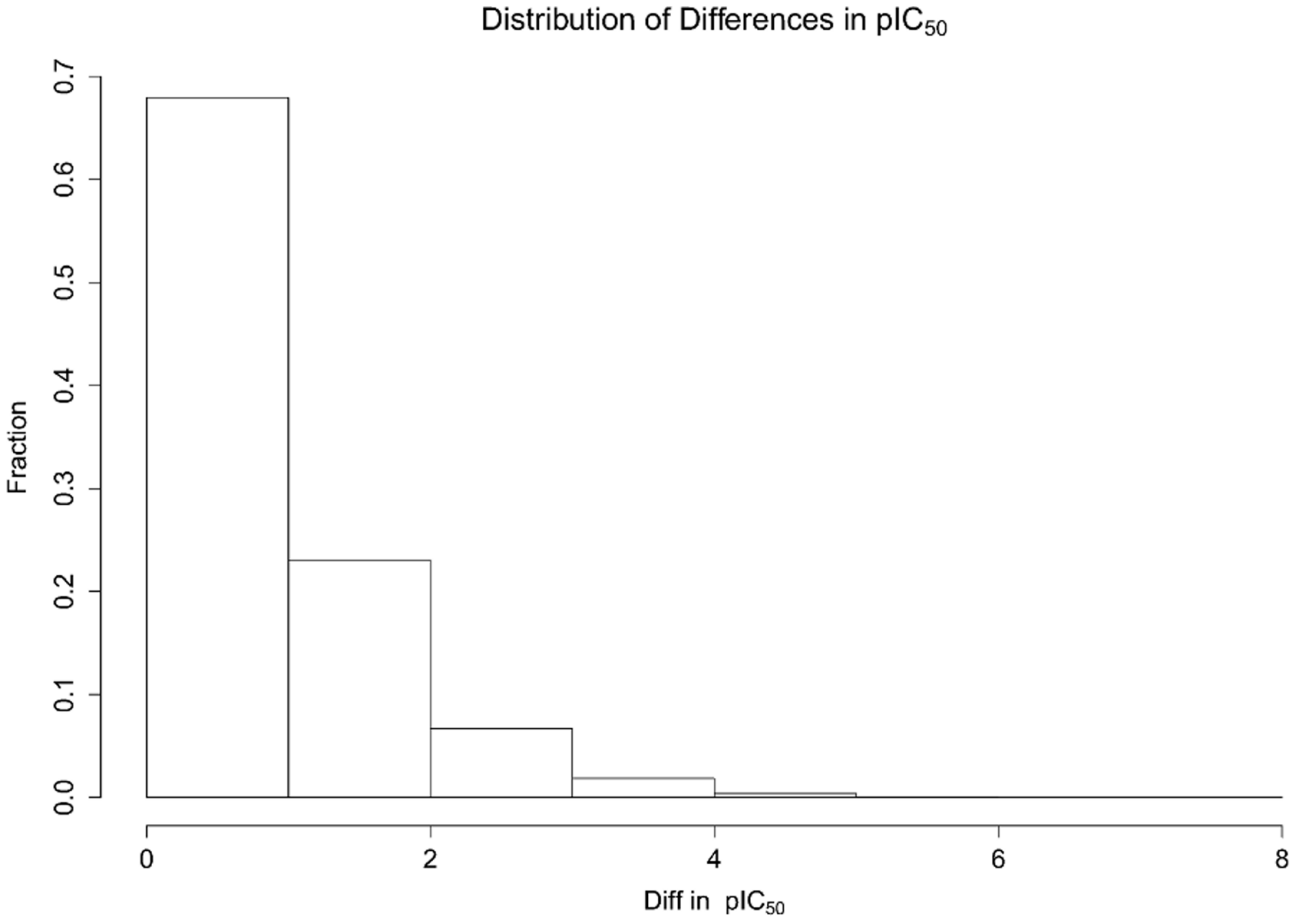
Distribution of the 16.844 pairs of ΔpIC_50_ values for protein-ligand systems with independent multiple measurements. The largest ΔpIC_50_ is 7.7 log units.

**Figure 3:**
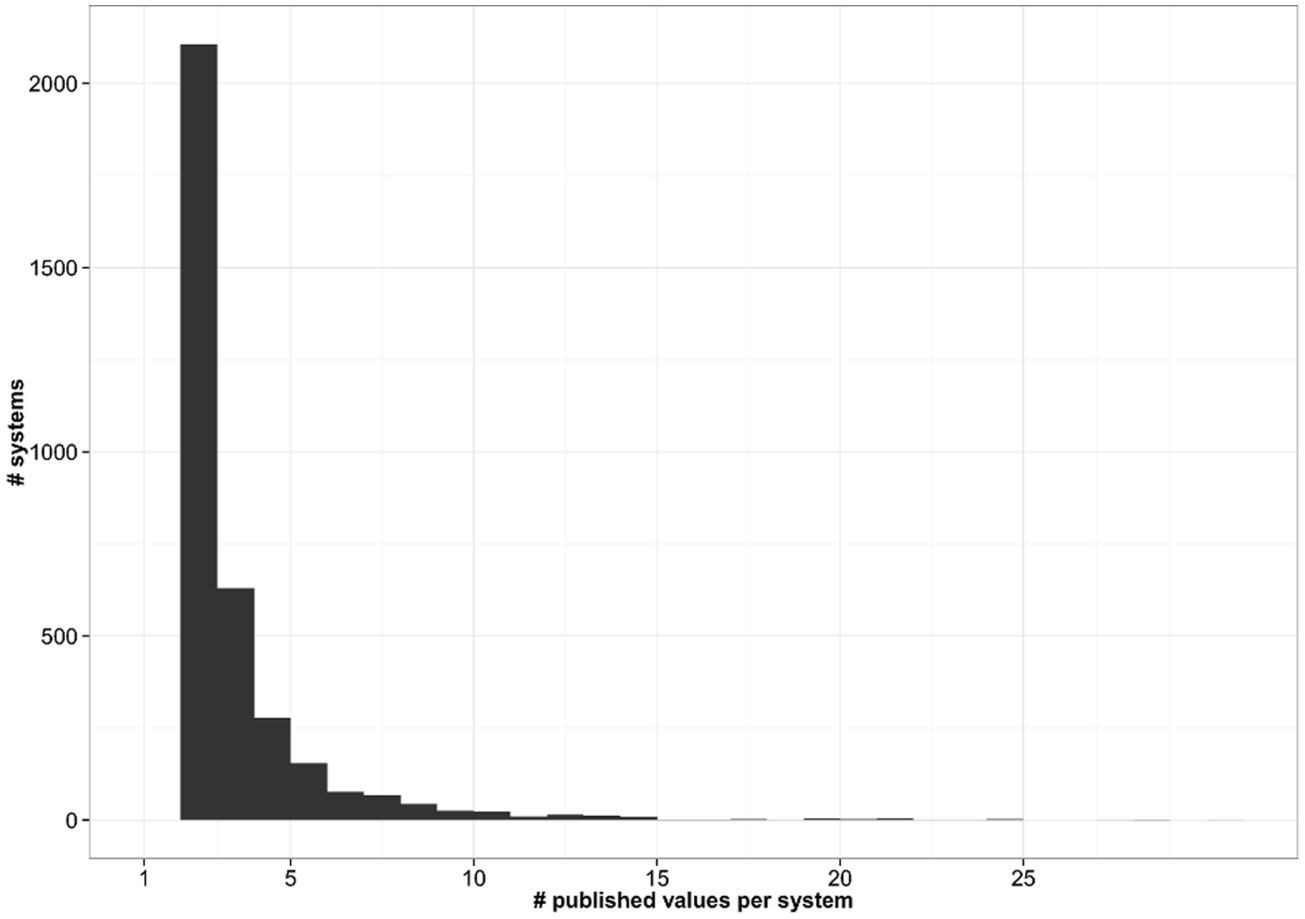
Number of published independent values per protein-ligand system.

The distribution of ΔpIC_50_ values and the distribution of the number of independent measurements per protein-ligand system are shown in Figures 2 and 3. Roughly 70% of all ΔpIC_50_’s are smaller than one log unit.

Most systems with multiple independent measurements have two or three independent measurements. The most frequently measured system is celecoxib on cyclooxygenase-2 with 30 independently measured IC_50_ values.

Sets of ten pairs of measurements for seven ranges of ΔpIC_50_ were closely inspected. The selected ranges of ΔpIC_50_ for the inspected ten cases span the whole range of ΔpIC_50_ (see Figure 2). The values of 3.2 and 1.1 were selected to avoid pairs which could contain combinations of citation of previous values and unit transcription errors. The findings are summarized in Table 2.

**Table 2:** Errors found for samples of pairs of measurements with specific differences in measured pIC_50_.

ΔpIC_50_	# invalid pairs out of 10	Error types found
From 4.7 to 7.8	9	unit error, receptor subtype error, stereochemistry error, cellular assay error
3.2	10	unit error, cellular assay error, target error, value error
2.5	8	unit error, receptor subtype error, value error
1.5	6 (+2 dubious)	unit error, cellular assay error, receptor subtype error, value error
1.1	1 (+2 dubious)	cellular assay error, receptor subtype error
0.05	1 (+1 dubious)	value error, different assay conditions
0.02	0 (+4 dubious)	original paper retracted, data cited from third source which is not available any more, receptor subtype error

We found that very high differences in pIC_50_ (ΔpIC_50_>2.5) were in most cases due to annotation errors. Some measurements had wrong units assigned (unit error). The receptor subtype was sometimes incorrectly assigned or not assigned at all (receptor subtype error). Other errors come from wrong stereoisomers of ligands (stereochemistry error), cellular assays assigned as biochemical assays (cellular assay error), incorrect target annotations (target error) and erroneous values extracted from original publications (value error).

Unit errors are the most common error. Receptor subtype errors occur most often for older publications (e.g., papers from the 1980’s with published IC_50_ values for dopamine receptors, opioid receptors, and mono-amino oxidases in general, i.e. without distinguishing the subtypes). This data is mixed with the subtype specific data in ChEMBL. Stereochemistry errors occur when the stereochemistry is wrongly extracted from the original literature. Cellular assay errors occur when the reported IC_50_ values have been measured in a cellular assay, despite being associated with a confident score greater than four (see Dataset preparation section).

Pairs with small ΔpIC_50_’s can also be composed of erroneously reported IC_50_ data. For example, the group of pairs with ΔpIC_50_ = 0.05 contains one case where the IC_50_ extracted from the literature is incorrect as in the original manuscript there is an activity range given, whereas in the ChEMBL database only one threshold of the range is reported with an equal sign. Another smaller set of problems come from retracted original publications (for example, the original publication [16], publishing an IC_50_ value for the compound with ChEMBL ID CHEMBL266497 on aldose reductase (CHEMBL2622), was retracted). Considering the number of invalid pairs out of the ten inspected for the seven ΔpIC_50_ ranges there is a high probability that pairs with ΔpIC_50_≥2.5 contains errors in the database or in the original publication.

**Figure 4:**
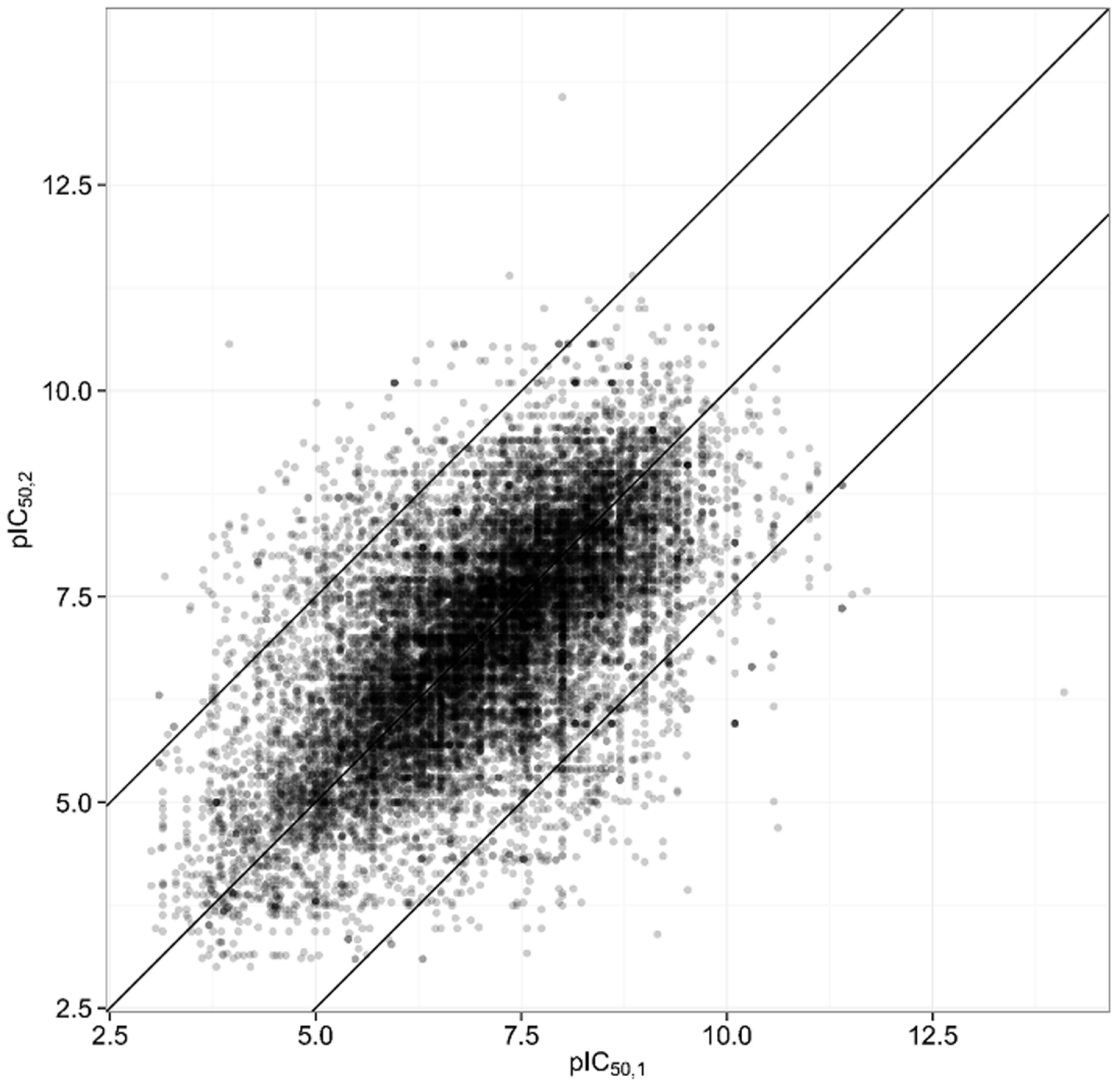
All Pairs of pIC_50_ values extracted from ChEMBL. The two outer diagonal lines indicate the 2.5 log unit threshold, outside which the probability for finding faulty pairs of measurements is very high. The extreme disagreements are all due to clear errors.

A plot of all pairs of pIC_50_ values is shown in Figure 4. The correlation coefficient for the raw extracted data is R^2^ = 0.40. Excluding a major part of the invalid pairs by removing all pairs with ΔpIC_50_≥2.5, the correlation coefficient becomes R^2^ = 0.53.

**Figure 5:**
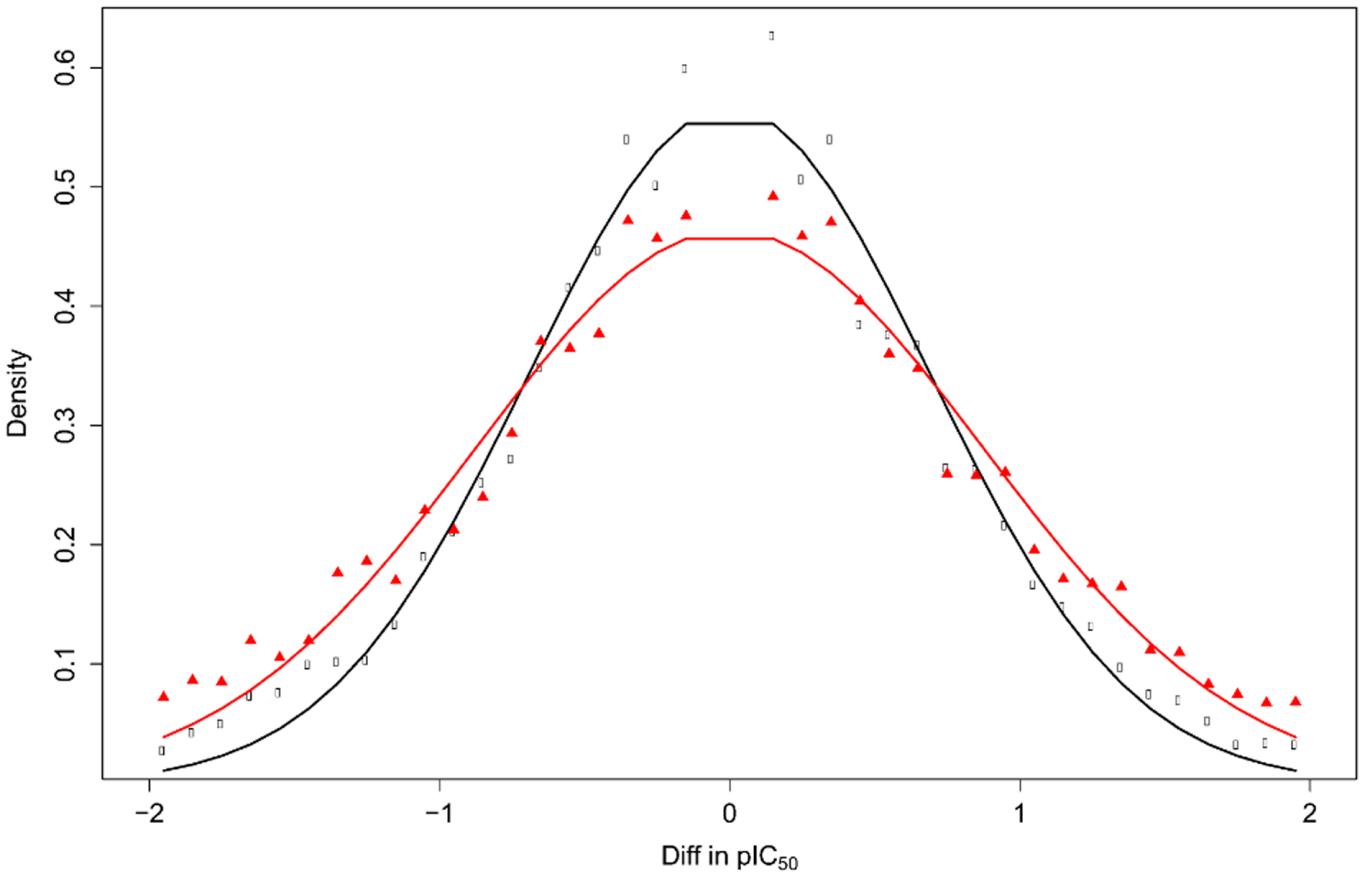
Fitted Gaussian distribution of ΔpIC_50_ (red) and ΔpK_i_ (black). The Gaussian distributions shown were fitted to all ΔpActivity values with an upper threshold ΔpActivity = 2.0. Standard deviations for the fitted Gaussian distributions are σ_pIC50_ = 0.87 and σ_pKi_ = 0.69. Note that since the σ here is calculated from pairs of measurements each containing experimental uncertainty and other sources of variability, it has to be divided by √2 in order to obtain the true σ of the individual measurements [13].

We also calculated the standard deviation σ of all ΔpIC_50_ and ΔpK_i_ values between 0.05 (lower threshold) and a variable upper threshold (1.5, 2.0 and 2.5) by fitting the data to a Gaussian distribution. The lower threshold of 0.05 was selected to remove pairs which were just rounded duplicates. The standard deviations obtained for the ΔpIC_50_ and ΔpK_i_ distributions are shown in Table 3. The fitted Gaussian and the raw distributions for ΔpIC_50_’s and ΔpK_i_’s with an upper threshold of 2.0 are shown in Figure 5.

**Table 3:** Standard deviation of a Gaussian distribution fitted to the inner part of the distribution of ΔpIC_50_ and ΔpK_i_.

Upper threshold	1.5	2.0	2.5
ΔpIC_50_	σ = 0.80	σ = 0.84	σ = 0.86
ΔpK_i_	σ = 0.66	σ = 0.68	σ = 0.68

The standard deviations of the ΔpIC_50_ data is constantly 21–26% larger than the standard deviation of the ΔpKi data. After dividing by √2, the σ for the Gaussian distribution fitted to all ΔpK_i_ values <2.5 then becomes 0.47 (a bit lower than the σ value of 0.54 previously calculated for heterogeneous pK_i_ data from ChEMBL version 12 data without upper threshold for ΔpKi data. [13] Since σ, MUE, and M_ed_UE are proportional to each other in Gaussian distributions, we can estimate σ, MUE and MedUE for the IC_50_ data to be 21–26% larger than the same metrics for pK_i_ data, yielding σ_pIC50_ = 0.68, MUE_pIC50_ = 0.55 and M_ed_UE_ pIC50_ = 0.43 (when using a factor of +25% for converting pK_i_ data to pIC_50_ data).

In order to test the alternative approach of directly obtaining quality metrics from the data, we calculated the quality metrics from the ΔpIC_50_ data with an upper threshold of ΔpIC_50_ = 2.5. Here, σ_pIC50_ = 0.68, MUE_pIC50_ = 0.54 and M_ed_UE_ pIC50_ = 0.43 are obtained. These values are very similar to the values obtained from comparing fitted Gaussian distributions and indicate that the erroneous pairs of measurements do not have a large effect on the overall result.

Similar performance was obtained considering only IC_50_ data with ChEMBL confidence score of nine (data not shown). As ChEMBL contains data from both human input and automatic extraction processes, we also looked if there was a difference between the two. Equally to the confidence score filtering, the results were similar with both data types.

**Figure 6:**
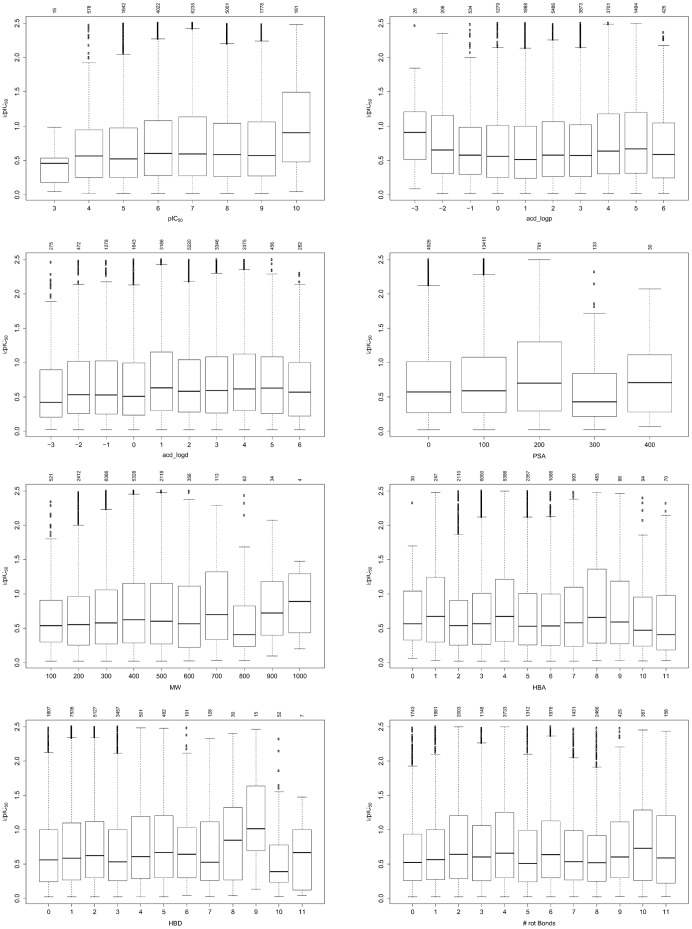
ΔpIC_50_ versus average pIC_50_ measured, logP, logD, polar surface area, molecular weight, number of hydrogen bond acceptors, number of hydrogen bond donors and number of rotatable bonds. The numbers above the boxplot indicate the number of ΔpIC_50_ values falling into the specific bin. Some boxplots are truncated at the very low and high ends because the low number of samples/bin makes the boxplot insignificant.

We checked whether the ΔpIC_50_ depends on the overall activity measured or on physicochemical ligand properties like logP, logD, molecular weight (MW), polar surface area (PSA), the number hydrogen bond acceptors (HBA), the number hydrogen bond donors (HBD) or the number of rotatable bonds. Boxplots of all those properties versus the ΔpIC_50_ are shown in Figure 6. The ΔpIC_50_’s depend neither on the average measured pIC_50_ nor on any of the ligand properties examined.

We also examined whether the ΔpIC_50_ depends on the combination of average activity and logP, since one might expect large deviations in measured pIC_50_’s for compounds with low activity and high logP due to solubility issues. Here we also did not find a clear trend (Figure S3).

### Can ChEMBL K_i_ and IC_50_ Data be Mixed?

**Figure 7:**
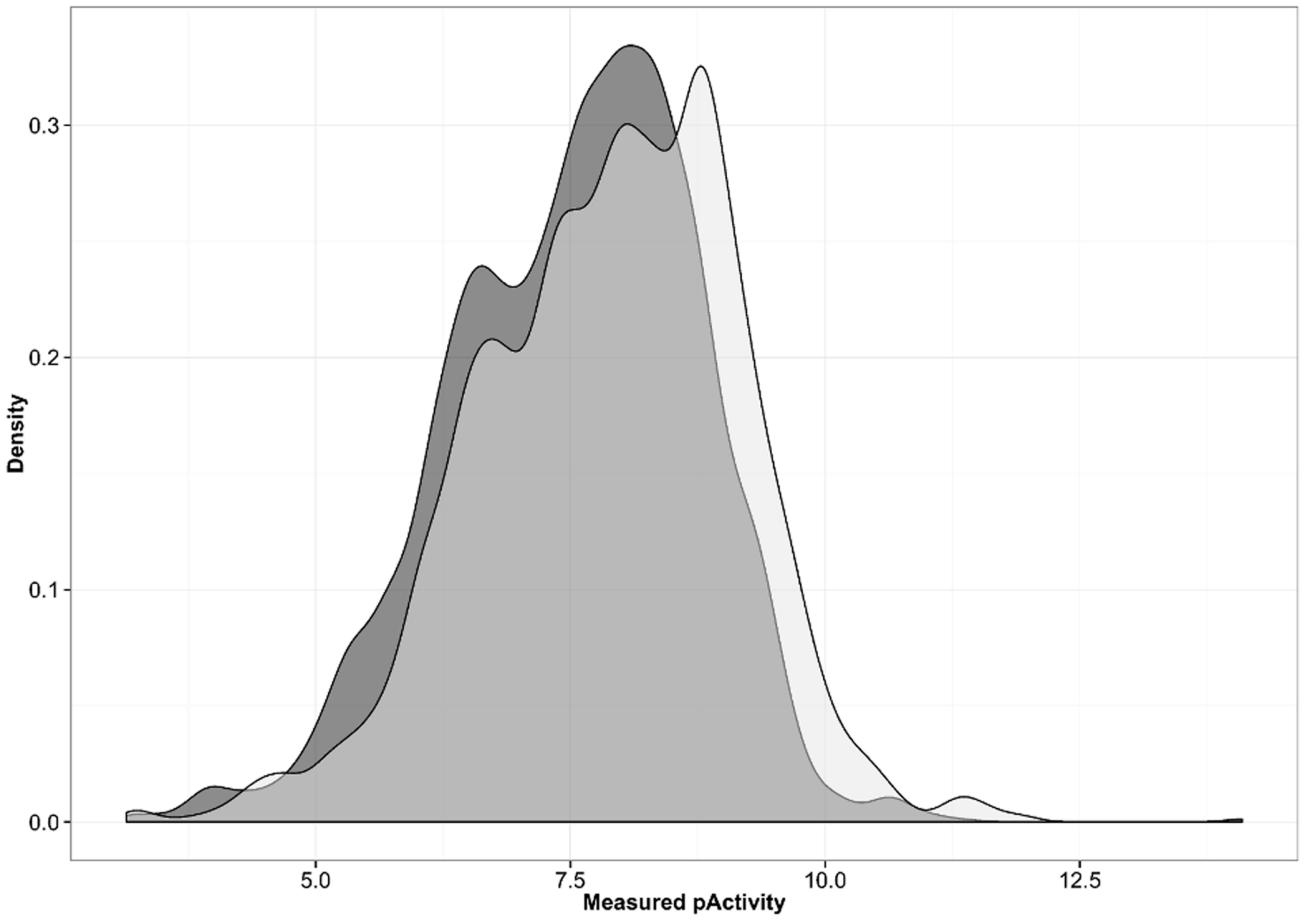
Distribution of published pIC_50_ (dark grey) and pK_i_ (light grey) values for protein-ligand systems with multiple independent measurements.

Empirical statistical models and SAR interpretations improve with the amount of data. Above, we have shown that the variability of heterogeneous IC_50_ data is roughly 25% worse than that of K_i_ data. Therefore it is not recommendable to add IC_50_ data to K_i_ data as this would lower the quality of the data. However, since there is much more IC_50_ data than K_i_ data available, it is interesting to see what happens by augmenting the IC_50_ dataset with additional K_i_ data. Figure 7 shows the distribution of pK_i_ and pIC_50_ data extracted from ChEMBL with the filters mentioned in Table 1. Overall, pIC_50_ and pK_i_ data show a similar distribution with the pK_i_ data slightly shifted towards higher values.

**Figure 8:**
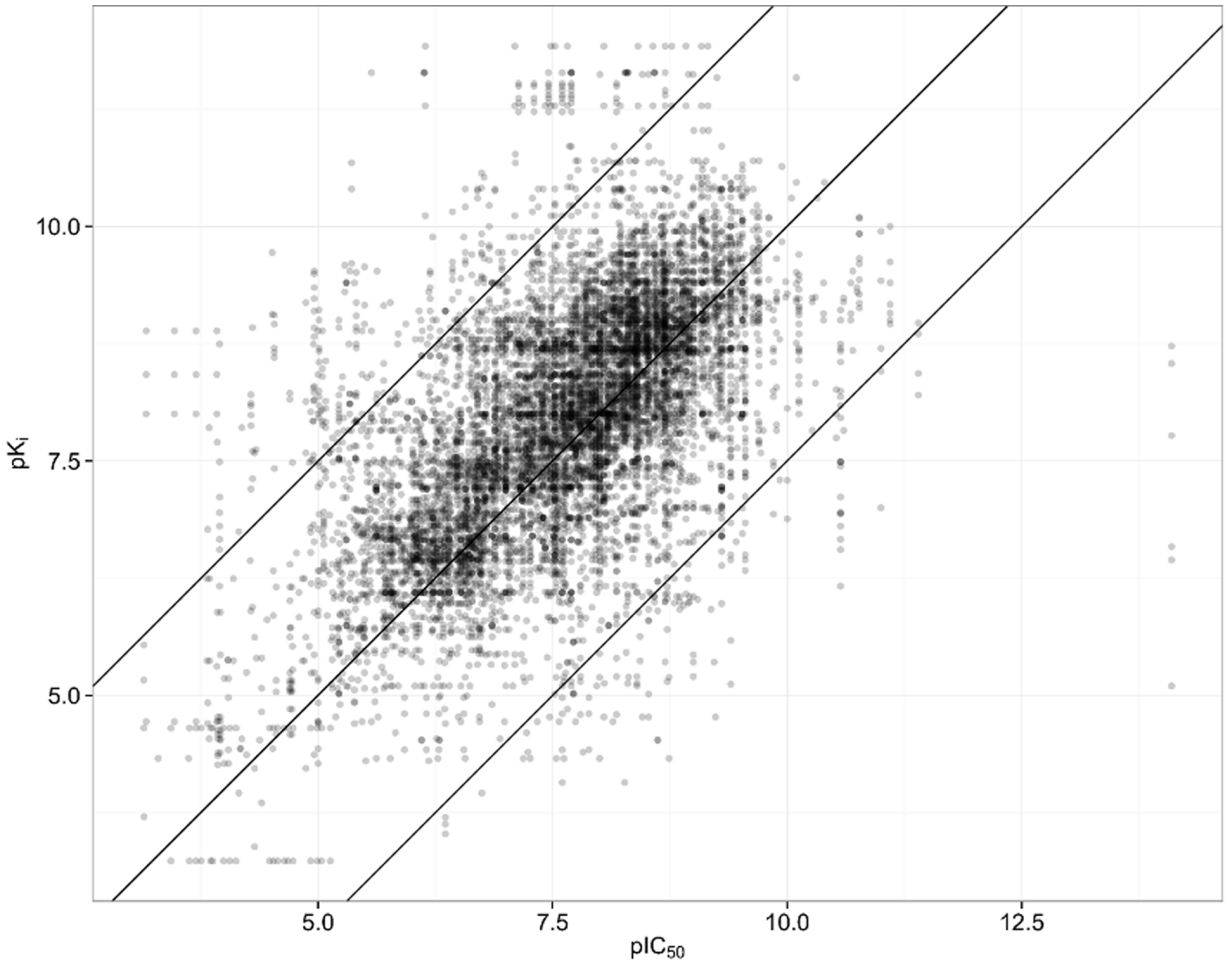
Measured pK_i_ versus measured pIC_50_ for identical protein-ligand systems.

For identical protein-ligand systems, we extracted all pairs of pK_i_ and pIC_50_ data that have passed the filters individually. This yields 11.556 pairs of measurements on 670 protein-ligand systems. A plot of measured pIC_50_ versus pK_i_ is shown in Figure 8.

Based on the Cheng-Prusoff equation and under the assumption of a competitive mechanism of action, pK_i_ values are larger or equal to pIC_50_ values. However due to unknown mechanism, experimental uncertainty and some database annotation errors in the data, there are a significant number of pairs where the pIC_50_ is larger than the pK_i_. On average, the measured pK_i_ values are 0.355 log units larger than the measured pIC_50_ values, corresponding to a factor of 2.3. A factor of 2 is in agreement with a balanced assay condition in which the substrate concentration is equal to the K_m_ value. This is often used in order to allow the detection of inhibitors with different mechanism of action.

After subtracting 0.35 log units from the pK_i_ values and correcting by √2, pK_i_ and pIC_50_ values agree with an R^2^ = 0.46, σ = 0.68, MUE = 0.54 and M_ed_UE = 0.43. The standard deviations of Gaussian distributions fitted to the inner part with an upper threshold of 1.5, 2.0 and 2.5 ΔpActivity units are 0.79, 0.83, and 0.85.

Overall, this is close to or even slightly better than the agreement obtained for pIC_50_ values with themselves. Therefore we can conclude that pK_i_ values can be used to augment pIC_50_ values without any loss of quality, if they are corrected by an offset. In the absence of assay information, the best guess for the conversion factor between K_i_ into IC_50_ is extrapolated from the average offset calculated from the heterogeneous ChEMBL data, i.e. a factor of 2.3, corresponding to 0.35 pActivity units.

## Discussion

In this contribution we show how the comparability of IC_50_ data can be analyzed using the public ChEMBL database. We find that when comparing all independently measured pIC_50_ data, the variability found for pIC_50_ data is approximately 25% larger than the variability found for pK_i_ data, with σ_pIC50_ = 0.68, MUE_pIC50_ = 0.55 and M_ed_UE_ pIC50_ = 0.43. These values correspond to the most probable variability of pIC_50_ data mixing from different (unknown) assays.

We want to stress that pIC_50_ data from different assays can only be compared under certain conditions. However, as discussed in the introduction, this is often done in large-scale data analysis. A standard deviation of 0.68 corresponds to a factor of 4.8, meaning that 68.2% of all IC_50_ measurements agree within a factor of 4.8, even when measured in different laboratories under potentially different assay conditions. One reason why the variability of IC_50_ data is found only moderately higher than the variability of K_i_ data might be that practically most of the IC_50_ assays may have been run using very similar assay protocols. Unfortunately, the assay descriptions available within ChEMBL are too terse to permit analyzing this any further.

**Figure 9:**
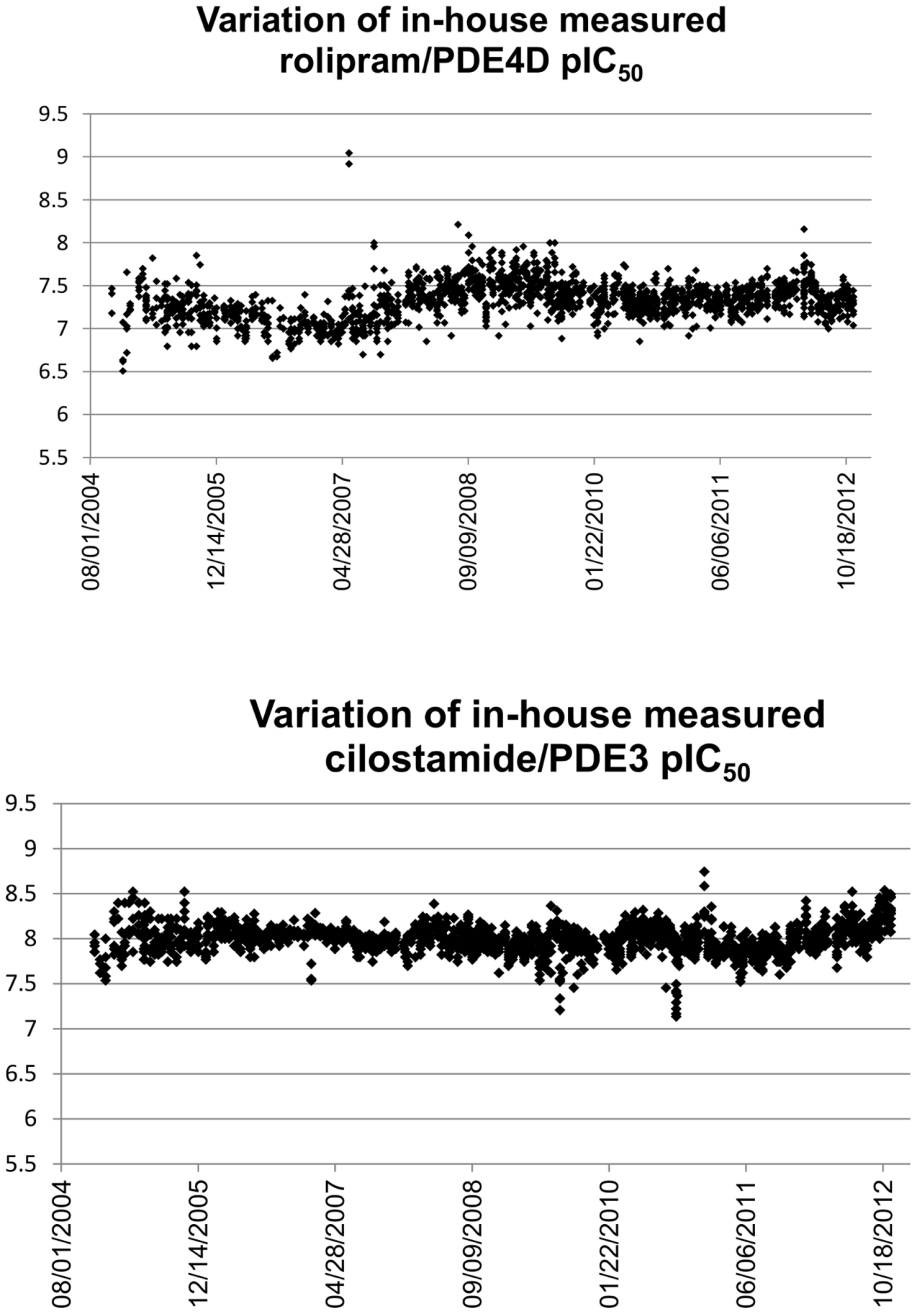
Variation of measured pIC_50_ values over time for rolipram/PDE4D and cilostamide/PDE3.

IC_50_ values measured in the same laboratory usually show a better reproducibility. From our in-house database, we extracted series of reference pIC_50_ values measured for assay standards. The plots in Figure 9 show the pIC_50_ values measured for rolipram on PDE4D and cilostamide on PDE3. The standard deviation of the pIC_50_ values are σ = 0.22 for rolipram/PDE4D and σ = 0.17 for cilostamide/PDE3.

There is some variation over time which could indicate changes in the assay conditions and solution handling. We also tried to find public series of at least ten compounds that have been measured in independent parallel assays. However, such series did not exist within ChEMBL as all the series we found were either measured in the same laboratory or the target protein was mistakenly annotated.

For extracting the pairs of IC_50_ data, which are indeed independently measured on the same protein-ligand system, we applied a set of filters that we have previously applied to filter and analyze K_i_ data. Here, the filters removed more than 90% of the IC_50_ data erroneously assumed to be independent measurements on the same protein-ligand system. When inspecting the remaining 20.356 pairs of measurements from 3.480 protein-ligand systems, we found that there are still a number invalid pairs, especially but not limited to the pairs with larger ΔpIC_50_. The main errors we found were unit transcription errors, wrong annotation of the receptor subtype, and annotation of cellular assays as biochemical assays. More rarely occurring errors were wrongly assigned stereochemistry, values and protein targets. These errors cannot be automatically detected and have to be manually curated out of the database over time [17].

In contrast to our previous study of K_i_ values, we observed a larger number of invalid pairs even for smaller ΔpIC_50_ approximately 2.5. To reduce the impact of these hard to find cases, we applied a different strategy to find the variability of the true pairs. By fitting a Gaussian distribution to the central part of the distribution we were able to compare the variability of the pIC_50_ data to the variability of the pK_i_ data. We found that the ratio between pK_i_ and pIC_50_ variability is relatively stable between 21 and 26% when varying the upper threshold for fitting the Gaussian distribution between 1.5 and 2.5 ΔpActivity units. Using this approach, we were able to estimate the variability of the IC_50_ data from the variability of the K_i_ data.

ChEMBL has a confidence score assigned for each activity value. The confidence score indicates how much the ChEMBL authors trust the value reported. Confidence scores below four indicate that the assay was a cellular assay, whereas confidence scores between four and nine indicate biochemical assays. In this study, we used all values that had a confidence score of at least four. The most confident data with a confidence score of nine was also exclusively used, but the results did not change. We also examined, whether there is a difference in data annotated as “autocurated” and data annotated as “expert” data. In this experiment, we also did not find any significant difference. The availability of assay description within ChEMBL would have allowed the analysis of whether specific assay types are statistically better comparable than other assay types or if the variability of pIC_50_ is lower in comparable assays. However, such information is not easily added to the database because this would require detailed assay ontologies and in the original literature assay details are often missing as well.

One might assume that higher IC_50_ values show a larger variability than for example single digit µM IC_50_ values because of solubility limits. However, our analysis shows that on the average this is clearly not the case. Moreover, the variability does not depend on any specific ligand properties such as logP, MW, PSA etc.

While the quality of pure K_i_ datasets would be reduced by adding IC_50_ data, we have shown that augmenting IC_50_ datasets by K_i_ data does not deteriorate the quality, if the K_i_ data is corrected by an offset. We found that pK_i_ values reported in ChEMBL are on average 0.35 log units higher than pIC_50_ values, which corresponds to a factor of 2.3. The IC_50_ to K_i_ conversion factor is exactly 2.0 in competitive monosubstrate IC_50_ inhibition assays, if the substrate concentration is set equal to its K_m_ value. This factor is close to the average difference between pKi and pIC_50_ values in ChEMBL and therefore in absence of any further specific assay knowledge available, a factor of 2.0 is the most probable conversion factor to convert K_i_ values to IC_50_ values.

## Summary and Conclusions

In this contribution, we present an analysis of the comparability of public heterogeneous IC_50_ data. We find that the agreement of independently measured biochemical IC_50_ values is only 23–30% worse than the agreement of pK_i_ data, irrespective to the used condition and type of assay. For heterogeneous biochemical pIC_50_ data, we find a variability with σ_pIC50_ = 0.68, MUE_pIC50_ = 0.55 and M_ed_UE_ pIC50_ = 0.43. Although theoretically IC_50_ values with different assay conditions should not be comparable, this is common practice in analyzing large-scale off-target and toxicity datasets. Our analysis quantitatively assesses the consequence in doing so. We believe that this knowledge should be important for everybody who decides to work with IC_50_ data from various heterogeneous sources. We also show that K_i_ data can be used to augment IC_50_ datasets without any loss of quality if corrected by a factor of 2, which is the conversion factor most frequently found by comparing the IC_50_/K_i_ values in ChEMBL for the same protein-ligand systems.

Nevertheless, public IC_50_ data extracted from ChEMBL14 is quite error prone. The most common errors we found are unit conversion errors, receptor subtype errors and errors in mixing up biochemical and cellular assay. The data quality is good enough to build large-scale fishing tools where errors partially cancel each other out, but for detailed SAR analysis and methods based on individual or very few data points like activity cliff or matched pair analysis it is mandatory to take recourse to the original literature and ensure that the values are correctly annotated and comparable.

This work augments our previous work where we focused on the experimental uncertainty of heterogeneous public K_i_ data. As we have previously stated, it is likely the data quality will rise over time by continuous iterative improvement of the large databases such as ChEMBL and BindingDB. In a different branch of affinity databases, smaller high-quality affinity databases, potentially combined with other physicochemical data or structural knowledge are being built up (see for example the CSARdock challenge [18], [19]). It will also be interesting to see what the reproducibility of such high-quality data is going to be.

It is surprising that we did not find in ChEMBL a single set of at least ten inhibitors for which IC_50_ values on the same target has been independently measured by different laboratories or a scientific contribution in literature addressing the comparison of heterogeneous IC_50_ values. Due to the scarcity of details about the experimental assay setup in both original publications and current large activity databases it is not possible to systematically analyze the comparability of the reproducibility of IC_50_ data for the same assay or various assay types under the same conditions. Using in-house data we were able to estimate the interlab reproducibility of IC_50_ for the same assay under the same conditions.

We hope that with this article we increase the awareness of noise added during mixing blindly public IC_50_ values during the data selection process for SAR analysis and QSAR models and its impact in limiting the maximal achievable performance of these techniques.

## Supporting Information

Figure S1 **Agreement of IC_50_ values for two dopamine transporter assays, measured in the same laboratory.** Here the pairs of measurements agree quite well with an R^2^ of 0.70 and a mean error of 0.29. According to the assay description of the primary literature, the assay conditions have been the same. The same is true for the norepinephrine transporter assay (R^2^ = 0.73, MUE = 0.29).
(DOCX)

Figure S2 **Agreement of IC_50_ values for two rattus norvegicus dihydrofolate reductase assays, measured in the same laboratory.** Although the assays have been run in the same lab on DHFR from the same species, the IC_50_ values of rattus norvegicus DHFR agree with R^2^ = 0.25 and MUE = 0.61.
(DOCX)

Figure S3 **Median ΔpIC_50_, binned according to average activity and logP.** The numbers indicate the number of entries per bin. We do not see a clear trend in this plot.
(DOCX)

Table S1 **All series where more than ten compounds have been measured in two parallel assays.**
(DOCX)

Text S1 **Closer inspection of Table S1.**
(DOCX)

Archive S1 **Python- and R-scripts to repeat the analysis.**
(GZ) Click here for additional data file.
