# Supplementary figures and images for: Comparability of Mixed IC50 Data – A Statistical Analysis
Source: PLoS One. 2013 Apr 16;8(4):e61007. doi: 10.1371/journal.pone.0061007 (PMC3628986; doi:10.1371/journal.pone.0061007)

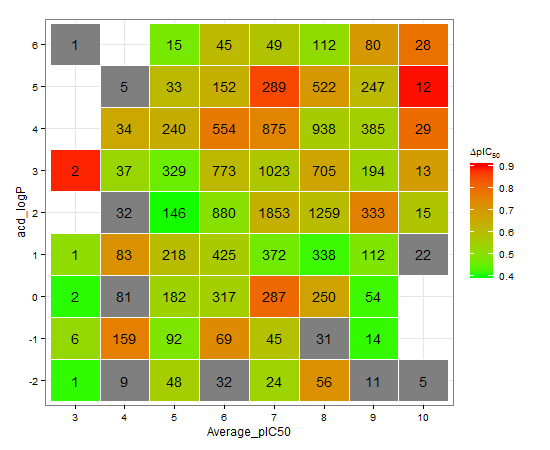

Supplement: Figure S3 — Median ΔpIC50, binned according to average activity and logP. The numbers indicate the number of entries per bin. We do not see a clear trend in this plot. (DOCX) [file pone.0061007.s003.docx]
