# Supplementary material for: Comparability of Mixed IC50 Data – A Statistical Analysis
Source: PLoS One. 2013 Apr 16;8(4):e61007. doi: 10.1371/journal.pone.0061007 (PMC3628986; doi:10.1371/journal.pone.0061007)
Supplement: Table S1 — All series where more than ten compounds have been measured in two parallel assays. (DOCX) [file pone.0061007.s004.docx]

| Target | CHEMBL  Target ID | Author overlap | Publication 1 | Publication 2 | R^2^ | MUE | # cpds |
| --- | --- | --- | --- | --- | --- | --- | --- |
| Dopamine Transporter | CHEMBL238 | Yes | J. Med. Chem. 2010, 53, 2204-14. | J. Med. Chem. 2009, 52, 6768-81. | 0.70 | 0.29 | 15 |
| Norepinephrine Transporter | CHEMBL222 | Yes | J. Med. Chem. 2010, 53, 2204-14. | J. Med. Chem. 2009, 52, 6768-81. | 0.73 | 0.29 | 11 |
| *Homo sapiens* dihydrofolate reductase | CHEMBL202 | Yes | J. Med. Chem. 2002, 45, 41-53. | J. Med. Chem. 1996, 39, 1271-80. | 0.37 | 1.07 | 23 |
| *Rattus norvegicus* dihydrofolate reductase | CHEMBL2363 | Yes | J. Med. Chem. 1995, 10, 1778-85. | J. Med. Chem. 1996, 39, 1271-80. | 0.25 | 0.61 | 11 |
| *Toxoplasma gondii* dihydrofolate reductase | CHEMBL2425 | Yes | J. Med. Chem. 1998, 41, 3426-34. | J. Med. Chem. 2005, 48, 1448-69. | 0.82 | 0.73 | 10 |
| hERG 1 | CHEMBL240 | Most Pro-bably | J. Med. Chem. 2002, 45, 3844-53. | Bioorg. Med. Chem. Lett. 2003, 13, 2773-5. | 0.80 | 0.35 | 11 |
| hERG 2 | CHEMBL240 | Most Pro-bably | Bioorg. Med. Chem. Lett. 2004, 14, 4771-7. | Bioorg. Med. Chem. Lett. 2003, 13, 2773-5. | 0.27 | 1.08 | 18 |
| hERG 3 | CHEMBL240 | Most Pro-bably | J. Med. Chem. 2009, 52, 4266-76. | Bioorg. Med. Chem. Lett. 2003, 13, 2773-5. | 0.94 | 0.10 | 14 |
| Nitric oxide synthase | CHEMBL3568 | Yes | J. Med. Chem. 2005, 48, 4783-92. | J. Med. Chem. 2002, 45, 2923-41. | 0.04 | 0.65 | 11 |
| Farnesyl diphosphate synthase | CHEMBL1782 | No | J. Med. Chem. 2008, 51, 2187-95. | J. Med. Chem. 2003, 46, 5171-83. | 0.87 | 1.05 | 13 |
| Norepinephrine Transporter 2 | CHEMBL222 | Yes | Bioorg. Med. Chem. Lett. 2008, 18, 4495-98 | Bioorg. Med. Chem. Lett. 2008, 18, 4491-4. | 0.74 | 0.33 | 11 |
| Serotonine Transporter | CHEMBL228 | Yes | Bioorg. Med. Chem. Lett. 2008, 18, 4495-98 | Bioorg. Med. Chem. Lett. 2008, 18, 4491-4. | 0.48 | 0.36 | 11 |
