# Supplementary material for: Comparability of Mixed IC50 Data – A Statistical Analysis
Source: PLoS One. 2013 Apr 16;8(4):e61007. doi: 10.1371/journal.pone.0061007 (PMC3628986; doi:10.1371/journal.pone.0061007)
Supplement: Text S1 — Closer inspection of Table S1. (DOCX) [file pone.0061007.s005.docx]

Upon closer inspection of the individual series in Table S1, it turns out that eight series had the following problems (highlighted in red):

- *Toxoplasma gondii* dihydrofolate reductase (DHFR): The data must have been wrongly transcribed into the database, since the later paper cites the earlier paper for the measured values.
- hERG 1: Both publications represent computational analyses (QSAR/ pharmacophore model) of previously published values. Although both publications cite the same sources for the data, different values are used.
- hERG 2: Both publications represent computational analyses (QSAR/ pharmacophore model) of previously published values. Although both publications cite the same sources for the data, different values are used.
- hERG 3: Both publications represent computational analyses (QSAR/ pharmacophore model) of previously published values. The data has been wrongly transcribed into ChEMBL. Using the correct data, all but one point have exactly the same value.
- Nitric oxide synthase: Both papers have identical authors, but the targets are wrongly annotated ion ChEMBL. The values from publication 1 are the measured affinity on isoform I-III together, whereas the values from publication 2 belong to the isolated isoform I.
- Farnesyl diphosphate synthase: Database problem: The assay organism assigned for both assay is *homo sapiens*, whereas according to the original literature the values from publication 2 have been measured on farnesyl diphosphate synthase from *Leishmania major*.
- Norepinehprine Transporter 2: Compound annotation error: One paper reports the affinities for the racemic mixtures, the other paper reports the affinities for the enantiopure compounds. Both measurement series have been carried out in the same lab by the same authors.
- Serotonine Transporter: Compound annotation error: One paper reports the affinities for the racemic mixtures, the other paper reports the affinities for the enantiopure compounds.

Both measurement series have been carried out in the same lab by the same authors.

In the end, four out of twelve pairs of series remain for comparison. All remaining pairs of series have been measured in the same laboratory and are therefore not independent. Overall, the four pairs of series originate from two different laboratories: The pairs of dopamine transporter and norepinephrine transporter come from one laboratory, and the pairs of human and rat dihydrofolate reductase come from another laboratory. A plot of the two measured values for the dopamine transporter is shown in Figure S1.
