# Supplementary material for: Comparability of Mixed IC50 Data – A Statistical Analysis
Source: PLoS One. 2013 Apr 16;8(4):e61007. doi: 10.1371/journal.pone.0061007 (PMC3628986; doi:10.1371/journal.pone.0061007)
Supplement: Supplementary file 5 [file pone.0061007.s002.docx]

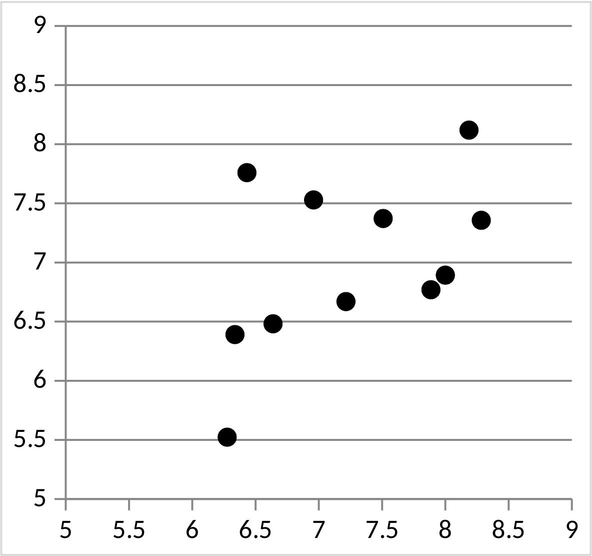
9
8.5
8
7.5
7
6.5
6
5.5
5
5
5.5
6
6.5
7
7.5
8
8.5
9
